# Reassessment of QTLs for Late Blight Resistance in the Tomato Accession L3708 Using a Restriction Site Associated DNA (RAD) Linkage Map and Highly Aggressive Isolates of *Phytophthora infestans*

**DOI:** 10.1371/journal.pone.0096417

**Published:** 2014-05-02

**Authors:** Ai-Lin Chen, Chu-Yin Liu, Chien-Hua Chen, Jaw-Fen Wang, Yu-Chen Liao, Chia-Hui Chang, Mong-Hsun Tsai, Kae-Kang Hwu, Kai-Yi Chen

**Affiliations:** 1 Department of Agronomy, National Taiwan University, Taipei, Taiwan; 2 AVRDC - The World Vegetable Center, Shanhua, Tainan, Taiwan; 3 Known-You Seed Co. LTD., Kaohsiung, Taiwan; 4 Institute of Biotechnology, National Taiwan University, Taipei, Taiwan; 5 Center for Biotechnology, National Taiwan University, Taipei, Taiwan; New Mexico State University, United States of America

## Abstract

Tomato late blight caused by the oomycete pathogen *Phytophthora infestans* (Mont.) de Bary is a major threat to tomato production in cool and wet environments. Intensified outbreaks of late blight have been observed globally from the 1980s, and are associated with migration of new and more aggressive populations of *P. infestans* in the field. The objective of this study was to reassess late blight resistance in the wild tomato accession L3708 (*Solanum pimpinellifolium* L.) against pathogens of different aggressiveness. An F_2:3_ genetic mapping population was developed using L3708 as the paternal parent. Two isolates of *P. infestans*, Pi39A and Pi733, were used for inoculation. Pi733 is a highly aggressive genotype that defeats three known late blight resistance genes, *Ph-1*, *Ph-2*, and *Ph-5t* in tomato. In contrast, Pi39A is a less aggressive genotype that defeats only *Ph-1*. Restriction site Associated DNA Sequencing (RAD-Seq) technology was used to massively sequence 90 bp nucleotides adjacent to both sides of *PstI* restriction enzyme cutting sites in the genome for all individuals in the genetic mapping population. The RAD-seq data were used to construct a genetic linkage map containing 440 single nucleotide polymorphism markers. Quantitative trait locus (QTL) analysis identified a new disease-resistant QTL specific to Pi733 on chromosome 2. The *Ph-3* gene located on chromosome 9 could be detected whichever isolates were used. This study demonstrated the feasibility and efficiency of RAD-Seq technology for conducting a QTL mapping experiment using an F_2:3_ mapping population, which allowed the identification of a new late blight resistant QTL in tomato.

## Introduction

Late blight, caused by the oomycete pathogen *Phytophthora infestans* (Mont.) de Bary, is a devastating disease affecting tomato (*Solanum lycopersicum* L.) and potato production especially in cool and wet environments. Intensified epidemic outbreaks of the disease have occurred throughout the world since the 1980s. This is associated with the migration of new and more aggressive pathogen populations [1], [2]. The global predominant genotype of *P. infestans* in both tomato and potato field before the 1980s was designated as a single lineage, US-1 [3]. Newer immigrated genotypes of *P. infestans* isolates are usually highly aggressive and resistant to metalaxyl fungicides; they quickly displace the original US-1 genotype [3], [4]. Dramatic population shift of *P. infestans* was occurred in Taiwan from 1998, and the highly aggressive isolate of the US-11 clonal lineage displaced the original US-1clonal lineage [5]. The two isolates, Pi39A and Pi733 used in this study represent the population shift of *P. infestans* in Taiwan. Pi39A belongs to the US-1 clonal lineage [5], whereas Pi733 belongs to the US-11 clonal lineage. They were a part of collections at AVRDC to survey *P. infestans* populations in Taiwan from 1997 to 2008.

Genetic factors associated with resistance to late blight in tomato have been characterized in several wild tomato species [6], [7], [8], [9], [10], [11]. Among these resistance genes, *Ph-3* has been widely used in tomato breeding programs as it confers resistance to *P. infestans* isolates in many regions [12], [13]. *Ph-3* was originally identified from the wild tomato *Solanum pimpinellifolium* accession L3708 and maps to the distal end of chromosome 9, close to the DNA marker TG591 [8], [14]. Two studies using advanced tomato lines derived from L3708, implied that in addition to *Ph-3* at least one other gene contributes to late blight resistance in L3708. The first study found that advanced lines containing the resistant *Ph-3* allele of L3708 were overcome in the field, but wild-type L3708 plants were not [15]. The second study demonstrated that one group of advanced lines conferred stronger resistance against highly aggressive *P. infestans* isolates than a different group of advanced lines, even though all lines had the same homozygous *Ph-3* genotype [12]. Therefore, it is important to determine whether there is a new genetic factor associated with resistance to late blight in L3708.

Quantitative trait locus (QTL) mapping has long been the standard method to identify resistance genes of wild tomato accessions against late blight [7], [8], [9], [10], [11]. Despite the development of genetic mapping populations and the measurement of phenotypes, typical QTL mapping requires great effort to identify new polymorphic markers and their genotypes, for all individuals in a mapping population, when new genetic crosses are made [8], [16], [17], [18], [19], [20], [21], [22], [23], [24], [25]. Restriction site Associated DNA sequencing (RAD-seq) [26] is a new sequencing-based genotyping method. It is able to overcome difficulties in identifying polymorphic markers, especially for crosses between accessions with low genetic polymorphism. The technical basis behind RAD-seq technology is to sequence a few million short DNA sequence reads anchoring at specific restriction enzyme sites in the genome. Alignment of sequencing reads between two parental genotypes allows detection of polymorphic single nucleotide polymorphism (SNP) sites as DNA markers, and identification of marker genotypes for all individuals in a mapping population, with or without the reference genome sequences [27]. Furthermore, RAD-seq reduces costs through multiplexing of bar-coded individuals [26]. RAD-seq technology has been successfully applied in numerous studies in crops requiring construction of genetic maps and QTL analysis [28], [29], [30], [31], [32], [33], [34].

This study reexamines the genetic components of resistance to late blight resistance in wild tomato *S. pimpinellifolium* L3708 by QTL mapping using RAD-seq technology. Our results demonstrated the feasibility and efficiency of RAD-Seq technology in conducting a QTL mapping experiment using an F_2:3_ mapping population in crops with reference genomic sequences, and identified a new late blight resistant QTL on chromosome 2 of L3708.

## Materials and Methods

### Plant materials

An F_2:3_ genetic mapping population was developed from the cross of *S. pimpinellifolium* accession L3708 (resistant parent) with the elite cultivar *S. lycopersicum* T3224 (susceptible parent). Four hundred and sixty nine individual F_2_ plants were grown in the field of the experimental farm of Known-You Seed Co LTD in Tainan, Taiwan for collection of F_3_ seeds. DNA was extracted from individual F_2_ plants for RAD-seq. One hundred and twenty F_2:3_ lines were randomly chosen to assess disease reaction by inoculation of two different *P. infestans* isolates. Six accessions, TS33 (*S. lycopersicum* L6161), TS19 (*S. lycopersicum* L6160), L3708 (*S. pimpinellifolium* L3708), LA1033 (*S. habrochaites* VI60017), WVa700 (*S. lycopersicum* var. *cerasiforme* L6193) and CLN2037B (*S. lycopersicum* AVTO9808) were used as differential hosts to identify physiological race of the two *P. infestans* isolates [5]. The tomato cultivar M82 was used as a susceptible control to assess disease severity for individual lines of the genetic mapping population [35].

Seeds were sown individually into 45 mm diameter plastic pots containing 1∶1 volume mixture of peat-lite (King Root Plant Medium #3, Taiwan) and peat moss. Liquid fertilizer (N:P:K = 30∶10∶10, HYPONEX #5, USA) was applied every other week. Seedlings were raised in a room at the Phytotron of the National Taiwan University at 25/20°C (day/night) for four weeks. They were then moved to a growth chamber for inoculation and symptom development.

### Assessment of disease reaction to late blight

*P. infestans* isolates Pi39A and Pi733 were used for the inoculation studies. Pi39A belongs to the US-1 clonal lineage and was re-isolated from Pi39-inoculated tomato plants at AVRDC [5]. Pi39 was originally collected in Tainan, Taiwan in 1997. In contrast, Pi733 belongs to the US-11 clonal lineage – a newly immigrated genotype, and was collected in Nantou, Taiwan in 2007. They were a part of collections at AVRDC to survey *P. infestans* populations in Taiwan from 1997 to 2008. The isolates were maintained on rye A agar plates at 18°C [36]. Inoculum was prepared from mycelial cultures grown on rye A agar plates at 16–18°C in the dark for 12–14 days. A sporangial suspension diluted to 5×10^4^ sporangia per milliliter was incubated at 12°C for 2 h to induce zoospore release. Plants were inoculated using a sprayer to atomize the zoospore/sporangia suspension onto the foliage to the point of run-off, and incubated at 20°C with 100% relative humidity in the dark for 24 h. Plants were then maintained at 60–95% relative humidity, with a daily 14 h light period.

Symptoms of late blight was visually scored based on a disease severity rating (DSR) of 0–6: where 0 indicates no symptoms; 1 indicates <5% leaf area affected and small lesions; 2 indicates 6–15% leaf area affected and restricted lesions; 3 indicates 16–30% leaf area affected and/or water-soaked flecks on stems; 4 indicates 31–60% leaf area affected and/or a few stem lesions; 5 indicates 61–90% leaf area affected and expanding stem lesions; 6 indicates 91–100% of leaf area affected, extensive stem damage, or a dead plant [8]. Plants were individually scored for DSR when the M82 susceptible control displayed the most severe symptoms.

To determine physiological race of each *P. infestans* isolate, 24 seeds from each of the 6 differential hosts and the susceptible control M82 were sowed, inoculated and evaluated together. One inoculation was carried out for each isolate. The mean of DSR score of a differential host was used to determine disease reactions against the inoculated *P. infestans* isolate. Disease reaction is designated as “resistant” when the mean DSR is less than 3. In contrast, disease reaction is designated as “susceptible” when the mean DSR is equal or larger than 3.

For the QTL mapping experiments, 6 plants of each of the 120 F_2:3_ lines, and 18 plants of the susceptible control M82 were evaluated together at each inoculation; four inoculation trials were carried out for each isolate. The mean DSR score of an F_2:3_ line inoculated with the same isolate was the average of the mean DSR scores from 4 trials and was used for data analysis in the QTL analysis. However, if less than 4 plants of an individual F_2:3_ line were germinated in a trial, no mean DSR score was calculated. The overall mean DSR score of an individual F_2:3_ line was treated as a missing value on the occasion that the mean DSR score were calculated less than 3 times out of 4 repeat trials.

### Construction of the RAD library

Total genomic DNA was isolated from young tomato leaves using a modified CTAB method [37], and further purified using the DNeasy Blood & Tissue Kit (QIAGEN, Venlo, Netherland) following manufacturer's instructions.

*PstI*-digested RAD libraries were prepared following the protocol of Etter et al. [38]. Sixty F_2_ samples were multiplexed with 60 different P1 barcodes in one RAD library (Table S1). For each sample, 1 µg gDNA was digested with 20 units of *PstI*-HF (New England BioLabs [NEB], Ipswish MA, USA) overnight in a 50 µL reaction volume. Samples were heat-inactivated for 20 min at 80°C. Digested DNAs were ligated to 2 µL 100 nM P1 bar-coded adapters, a modified Solexa adapter (Table S1), along with 1 µL 10× NEBuffer4 (NEB, Ipswish MA, USA), 0.5 µL 2000 unit µL^−1^ T4 DNA ligase (NEB, Ipswish MA, USA), and 0.6 µL 100 mM riboATP (Promega, Madison WI, USA) in a 60 µL reaction volume for 1 h. Samples were heat-inactivated for 20 min at 65°C. Samples were pooled (20 µL each, 60 samples) and a 50 µL aliquot was loaded into a 0.5 mL PCR tube (Axygen catalog # PCR-05-C, Corning, Tewksbury, MA, USA). DNAs were sheared using a Bioruptor UCD-200 sonicator (Diagenode, Liège, Belgium) set to high, for 3 runs of 7 min (30 s on/30 s off). The peaks of most DNA aliquots were approximate 300 bp. If the peak of sheared DNA was over 500 bp, then additional sonications were performed until the peak became less than 500 bp. Sheared DNA aliquots were pooled and concentrated using two MinElute columns (QIAGEN, Venlo, Netherland) and eluted with 40 µL EB buffer (10 mM Tris-Cl, pH8.5) in each column. Eluted DNAs were combined and size selected using Agencourt AMPure XP magnetic beads (Beckman Coulter, Brea CA, USA) with a volume DNA:beads ratio of 1∶0.65; this removed DNA fragments of less than 300 bp [39]. Recovered DNAs were suspended in 20 µL EB buffer, and treated using a Quick Blunting Kit (NEB, Ipswish MA, USA) for end repair. 1 µL Blunt Enzyme Mix, 2.5 µL 10× Blunting Buffer, and 2.5 µL 1 mM dNTP mix were added to the 20 µL DNA solution. The mixture was incubated at 25°C for 30 min. Agencourt AMPure XP magnetic beads (Beckman Coulter, Brea CA, USA) were used for reaction clean-ups with a volume DNA:beads ratio of 1∶1.8; this removed DNA fragments of less than 50 bp. The repaired dsDNAs were suspended in 20 µL buffer EB and quantified using the Quant-iT dsDNA HS Assay Kit (Life Technologies, Carlsbad CA, USA). Adenine was added to the 3′ ends of dsDNA fragments in a 50 µL reaction volume containing 1 µg dsDNAs, 5 µL 10× NEBuffer2, 1 µL 10 mM dATP, and 3 µL of 5 unit µL^−1^ Klenow Fragment (NEB, Ipswish MA, USA) mixed and incubated at 37°C for 30 min. DNAs were cleaned using 90 µL (1.8× volume) Agencourt AMPure XP magnetic beads (Beckman Coulter, Brea CA, USA), and suspended in 45 µL EB buffer. Reactions for P2 adapter ligation were assembled by adding 1 µL 10 µM P2 adapter (Table S1) to the dsDNA solution along with 5 µL 10× NEBuffer2, 0.5 µL 100 mM riboATP (Promega, Madison WI, USA), and 0.5 µL 2000 unit µL^−1^ T4 DNA ligase (NEB, Ipswish MA, USA). The mixture was incubated at 20°C for 3 h. The P2 adapter-ligated dsDNAs were then purified using 35 µL (0.7× volume) Agencourt AMPure XP magnetic beads (Beckman Coulter, Brea CA, USA), suspended in 20 µL EB buffer, and quantified using a Quant-iT dsDNA HS Assay Kit (Life Technologies, Carlsbad CA, USA). 50 ng of this DNA product was PCR amplified using 4 µL 10 µM modified Solexa primer mix (Table S1) and 50 µL Phusion High-Fidelity PCR Master Mix (NEB, Ipswish MA, USA) in a 100 µL reaction volume. The PCR setting was 98°C for 30 s, followed by 18 cycles of 98°C for 10 s, 66°C for 30 s, 72°C for 30 s, and a final extension reaction at 72°C for 5 min. The PCR-enriched product was purified with 70 µL (0.7× volume) Agencourt AMPure XP magnetic beads (Beckman Coulter, Brea CA, USA), and normalized to 10 ng µL^−1^. One RAD library was sequenced in one lane of an Illumina Hiseq2000 flow cell (100 bp single-end reads) (Illumina Inc., San Diego, CA, USA). Next-generation sequencing (NGS) was provided by the Genome Research Center at the National Yang-Ming University, Taiwan. 411,738,303 reads were obtained (Table S2). Sequences are available at the Sequence Read Archive http://www.ncbi.nlm.nih.gov/Traces/sra/, at accession SRA144571 (Table S2).

### Sequence analysis

Stacks v1.08 (http://creskolab.uoregon.edu/stacks/) [27] and CLC Genomics Workbench v6.5.1 (CLC Bio, http://www.clcbio.com) were used for sequence analysis. Raw sequencing reads were processed using the “process_radtags” command in Stacks to filter out reads with quality scores less than Q10, and to sort reads to individual samples based on barcode sequences. Sorted reads for each sample were aligned to the tomato genome sequence build SL2.40 (SOL Genomics Network, http://solgenomics.net/), using the read mapper tool in the CLC Genomics Workbench. Stringent parameters were used to prevent high false positive rates for SNP calling. As most RAD read sequences had low possibility of overlap, the parameters for sequence alignment were set to allow no more than two mismatches for the 90 bp short reads (length fraction  = 1.0, similarity fraction  = 0.97), and to discard aligning results if reads were mapped to more than two positions in the genome. The other parameter settings for read mapping were mismatch cost  = 2, insertion cost  = 3, and deletion cost  = 3. The sequencing-read-alignment files of the two parents were used to define the SNP sites, and those of the 120 progenies used to determine genotypes at the defined SNP sites. This was performed using the “ref_map.pl” command of the Stacks software, set at default parameters, except for minimum read depth, which was set to 3.

### Development of additional SNP markers for genotyping by VeraCode technology

In order to obtain independent genotypic data different from the RAD-seq data to delimit the chromosomal region where recombination events were suppressed, 114 SNP sites between the two parental lines were successfully developed as a customized genotyping panel using VeraCode technology (Illumina Inc., San Diego, CA, USA) (Table S3). At beginning, a total of 144 candidate SNP sites were chosen from a SNP database that was independently built from the RAD-seq data. The SNP database contains approximately 0.7 million SNPs distributed evenly in the tomato genome and was obtained by a comparison between two assembled genomic sequences of the parental lines, *S. pimpinellifolium* L3708 and *S. lycopersicum* T3224, each of which was subjected to whole genome shotgun sequencing with approximately 8× coverage. To select the candidate SNP sites for Veracode SNP markers, two adjacent SNP sites were chosen with an approximately physical distance of 6 Mb on each of the 12 tomato chromosomes. Genotypes of the 144 VeraCode SNP markers for individuals in the F_2:3_ mapping population were determined using the GoldenGate Genotyping Assay on the BeadXpress Reader System (Illumina Inc., San Diego, CA, USA) [40]. Fluorescence signal data were analyzed using GenomeStudio genotyping module v1.0 (Illumina Inc., San Diego, CA, USA). Sixteen markers failed to generate genotypes, and a further 14 markers showed no polymorphism in the mapping populations. Reproducibility of genotypes of the VeraCode SNP markers can reach to 99.9%. It hence provide great accuracy to delimit the recombination suppression regions. A substantial number of RAD markers located in these chromosomal regions were able to be ignored, so it greatly enhanced the efficiency to run the JoinMap software which is used to construct the genetic linkage map.

### Genetic map construction, and QTL analysis

JointMap v4.1 software was used to construct genetic maps [41]. Regression mapping was used as the mapping algorithm. A linkage group was defined when markers showed recombinant frequency smaller than 0.4, and independence LOD values larger than 7.0. The Haldane mapping function was used to calculate genetic distance [42].

MapQTL v6 software was used for QTL analysis [43]. Interval mapping was first used to identify markers that significantly associated with phenotypic variations in the mapping population. Next, the MQM mapping module in the MapQTL software (similar to composite interval mapping) was used to confirm the final result of the QTL analysis by iterating significant cofactor (marker) selection. A regression algorithm was used to calculate the approximate LOD scores for interval mapping, MQM mapping, and the permutation test. The mapping step size was set to 1.0 cM. The empirical threshold of LOD score to claim QTLs was set to 4.0; this was obtained by running the permutation test 50,000 times.

## Results

### Designation of physiological races of *Phytophthora infestans* isolates

Pi733 (US-11 clonal lineage) and Pi39A (US-1 clonal lineage) isolates were used in this study to investigate the resistance profile of L3708 against different clonal lineages of *P. infestans*. Designation of physiological race for these two isolates was based on the disease reaction of six differential tomato hosts: TS19 (*ph+*), TS33 (*Ph-1*), WVa700 (*Ph-2*), CLN2037B (*Ph-3*), L3708 (*Ph-3,4t*), and LA1033 (*Ph-5t*) [5]. Pi39A only infected TS19 and TS33, and was designated as race T1. Pi733 infected TS19, TS33, WVa700, and LA1033, and designated as race T1,2,5t (Table 1).

**Table 1:** Race designation for *Phytophthora infestans* isolates Pi39A and Pi733.

differential host		*Phytophthora infestans* isolates			
line (resistance gene)		Pi39A		Pi733	
		DSR^a^	response^b^	DSR^a^	response^b^
M82		6.00	S	6.00	S
TS19	(*ph+*)	6.00	S	6.00	S
TS33	(*Ph-1*)	6.00	S	6.00	S
WVa700	(*Ph-2*)	1.00	R	6.00	S
CLN2037B	(*Ph-3*)	0.93	R	0.85	R
L3708	(*Ph-3*,*4t*)	0.30	R	2.38	R
LA1033	(*Ph-5t*)	0.39	R	5.03	S
designated race		T1		T1,2,5t	

a DSR: disease severity rating.

b R:resistance; S:susceptible.

### Phenotypic evaluation of disease reaction for the mapping population

**Figure 1:**
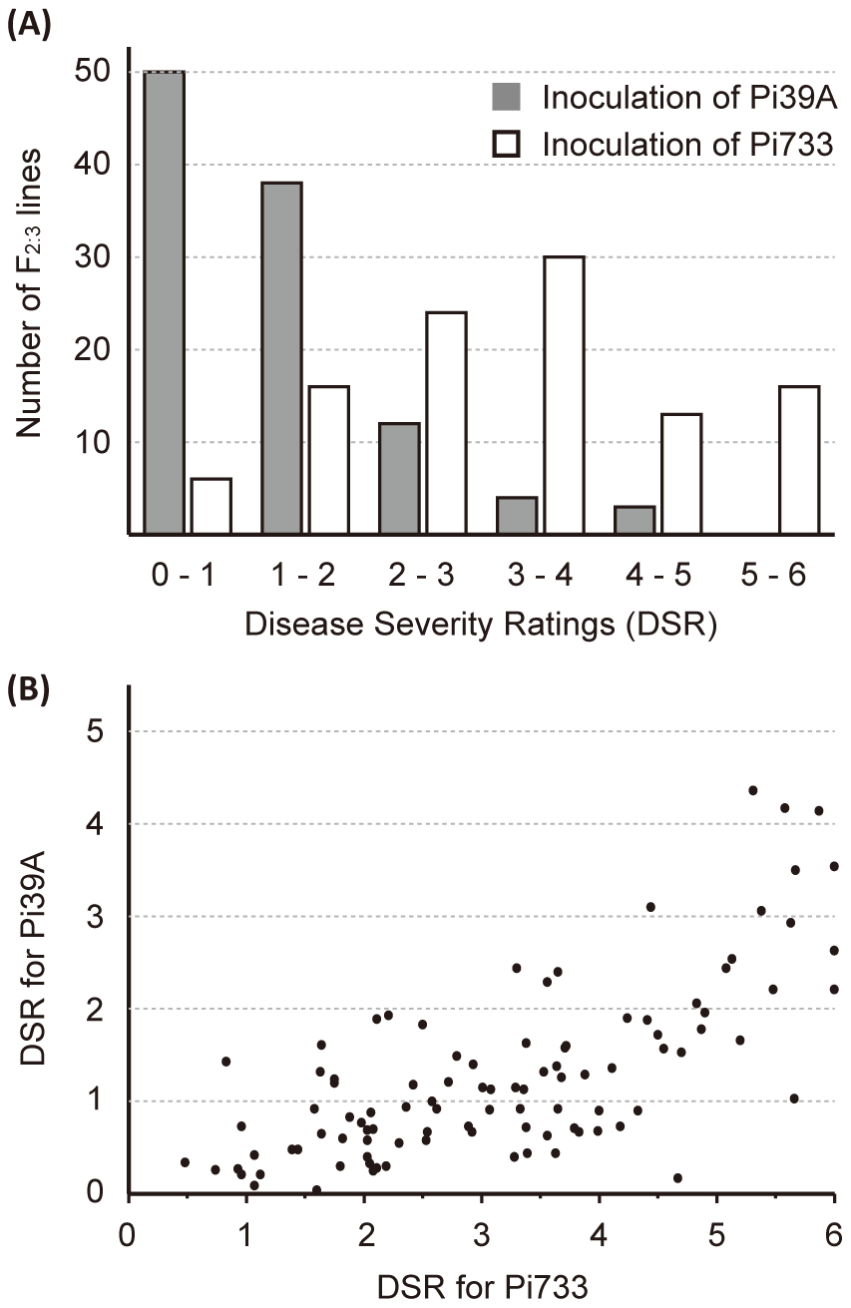
Disease severity rating (DSR) for isolates Pi39A and Pi733. (A) Distribution of DSR in the mapping population. (B) Relationship of the two DSR score sets generated from independent inoculations of Pi39A and Pi733.

To identify resistance QTLs to different *P. infestans* isolates, an F_2:3_ genetic mapping population was developed from a cross of resistant *S. pimpinellifolium* accession L3708 and susceptible cultivated tomato *S. lycopersicum*. One hundred and twenty F_2:3_ lines were randomly selected for phenotypic evaluation. Seedlings grown from the 120 F_2:3_ lines were inoculated separately with either Pi39A or Pi733 isolates. Disease severity ratings for each inoculation were scored at the time that the susceptible tomato cultivar M82 control reached maximum severity. The DSR scores for resistant parent L3708 were 0.112 and 0.144, for Pi39A and Pi733, respectively. The DSR scores of the 120 F_2:3_ lines against Pi39A ranged from 0.109 to 4.984, with a mean of 1.537, and skewed towards zero (Figure 1A; Table S4). In contrast, DSR scores of the 120 F_2:3_ lines against Pi733 ranged from 0.346 to 5.921, with a mean of 3.149, and showed an approximate equal frequency among the different DSR classes (Figure 1A; Table S4). These results implied that the Pi733 isolate was more aggressive than the Pi39A isolate. The correlation coefficient between the DSR scores when inoculating Pi39A and Pi733 was 0.704 (Figure 1B), which implied that common genes conferred resistance against both isolates.

### Genotyping and construction of genetic map

The total read number of the 120 bar-coded F_2_ samples obtained from two lanes of the Illumina Hiseq2000 platform was 396,100,974. Approximate 92% of reads passed the default quality filter in the Stack software, and 76% of reads aligned to the tomato genome sequence build SL2.40. The aligned read numbers of individual samples in the F_2_ population ranged from 842,457 to 4,446,219, with an average of 2,504,121 (Table S2). The RAD reads of the two parental samples were generated from an additional RAD library. There were 5,925,802 and 6,384,883 aligned reads for the resistant parent *S. pimpinellifolium* accession L3708 and the susceptible parent *S. lycopersicum*, respectively. A total of 67,339 distinct 90 bp DNA sequences on the tomato genome sequence build SL2.40 were aligned with RAD reads from both parents, and 12,718 sites of 90 bp sequences were defined as RAD markers showing distinct homozygous haplotypes between the two parents. The genotypes of these RAD markers for each of the 120 F_2_ samples were determined, however, a substantial portion of genotypes for all RAD markers were missed. To remove potentially problematic RAD markers from use for constructing the genetic map, three artificial criteria were set: (1) markers with more than 10 missing data points were removed; (2) for the goodness-of-fit test of unbiased genotype segregating ratio, markers with a chi-square value larger than 30 were removed; (3) markers not mapping with reference sequences of the 12 chromosomes were removed. After filtering, 4697 RAD markers remained. For ease of identification, the RAD markers were designated using a “00g00000000” format. The first two digits indicate the chromosome number, while the last eight digits indicate the physical position of the *PstI* cutting site on tomato genome reference sequence build SL2.40.

**Figure 2:**
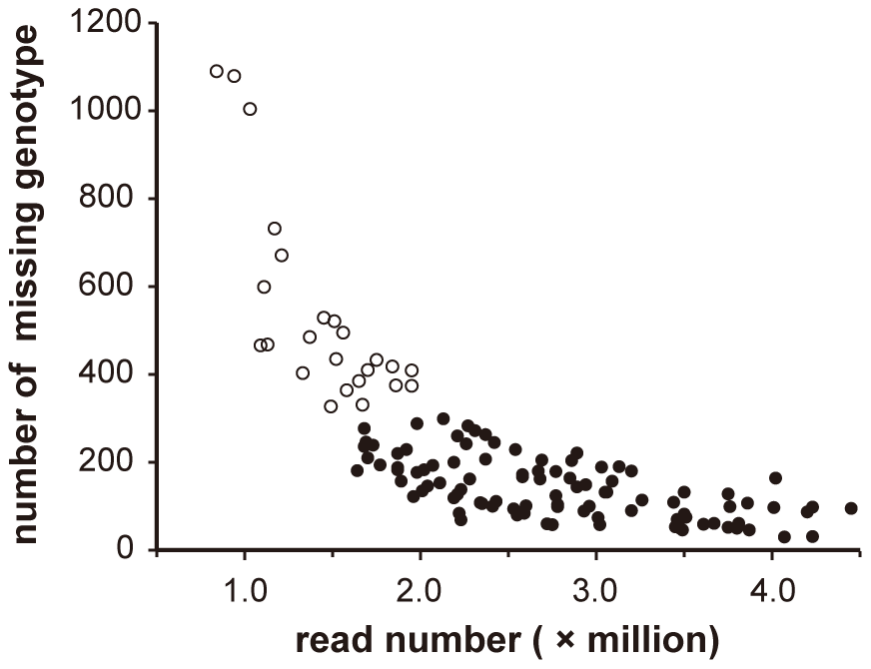
Relationship between read number and missing data. Circles indicate each line of the F2 mapping population. The 96 filled circles represent samples used for genetic map construction, while the 24 open circles represent samples removed because of a high number of missing data in the population.

The nature of RAD markers tends to produce low proportions of miscalled genotypes; these mainly result from insufficient read depths of a defined RAD marker [44]. To mitigate genotyping error, 24 F_2_ samples were removed using the number of missing genotype as the selection criterion. Most removed samples also contained a lower number of RAD reads (Figure 2). Genotypes of the remaining 96 F_2_ samples were used to construct the genetic map. Additional 94 genotypes belonging to customized VeraCode SNP markers were added to the genotypic data. This allowed precise delimitation of chromosomal regions where very low recombination occurred for each of the 12 linkage groups. Among the 4697 RAD markers, 2047 contained no more than 2 missing genotypes out of the 96; these were selected for linkage analysis, along with the 94 VeraCode SNP markers, using JoinMap software. From the linkage analysis, the 2141 markers were separated into 12 linkage groups using a LOD threshold parameter of 7.0. Markers with physical locations identified on the same chromosome were grouped into the same linkage group, except for marker 06g33932831, which was grouped with markers on chromosome 9. The cause of this exception remains to be determined.

To construct a concise accurate linkage map, only a part of the RAD markers were selected. The selection criteria included: (1) allow the order of markers on the genetic map to be the same as the order on the physical map; and (2) the genetic distance between adjacent markers was in the range of 2 to 5 cM if a suitable marker was available. To achieve both criteria systematically, RAD markers from each linkage group were selected by the following strategy. Markers were ordered by physical position on the color-coded genotype panel in the JointMap program. Possible genotyping errors were identified by looking for individual genotypes different from neighboring marker genotypes. Crossover events could be identified when one genotype the same for more than three adjacent markers along the marker order switched to a different genotype identical for the following three markers in the same individual. The genotyping errors of each marker were counted, and crossover events along the marker order were recorded for the whole mapping population. In recombination blocks defined by four to seven crossover events, the marker with the lowest number of possible miscalling genotypes was selected.

**Figure 3:**
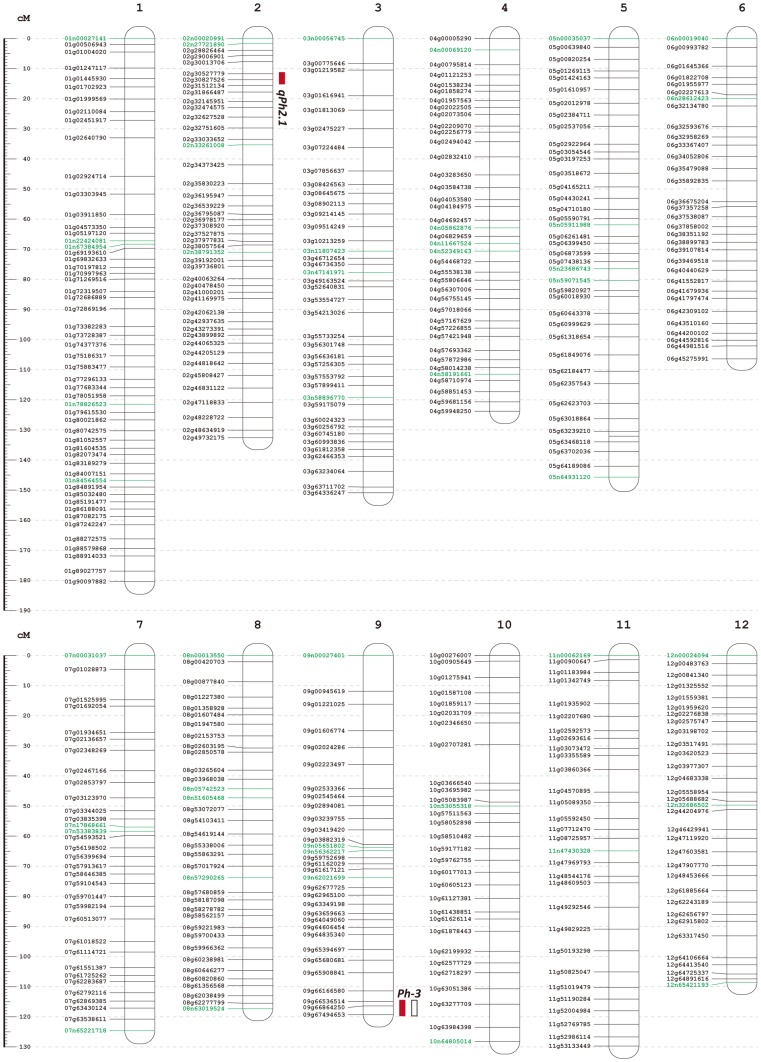
Genetic map and results of QTL analyses for resistance to tomato late blight. The naming of the RAD markers was in the format “00g00000000”. The first two digits indicate chromosome number, while the last eight digits indicate the physical position of the *PstI* cutting site on the tomato genome reference sequence build SL2.40. The naming of the VeraCode SNP markers was designated in the same way as the RAD markers, except for replacing “g” with “n”, and the last eight digits indicating the physical position of the SNP site. VeraCode SNP markers names are highlighted green. The red bar indicates the location of the QTL resistant to isolate Pi733, and the hollow bar indicates the location of the QTL resistant to isolate Pi39A.

The final genetic map for the 12 chromosomes included 395 RAD markers and 45 VeraCode SNP makers (Figure 3; Table S5 and S6). The average genetic distance between two adjacent markers was 3.56 cM. None of the adjacent marker pairs had genetic distances larger than 13 cM. This indicated that the marker density of the whole genome was sufficient to capture major genetic effects causing phenotypic variations for QTL analysis.

### QTL analysis

To understand whether the wild tomato accession L3708 confers additional genes resistant to highly aggressive isolates of *P. infestans*, phenotypic data collected from inoculation of Pi39A and Pi733 were used separately for QTL analysis. Because the same genetic materials and genotypic data were shared for different QTL analyses, comparison between two QTL analyses could reveal distinct genetic factors contributing to new resistance against the highly aggressive isolates of *P. infestans* Pi733.

A QTL at the distal end of chromosome 9 was detected when the Pi39A isolate was used (Figure 3; Table 2 and S7). The location of this QTL is the same as the *Ph-3* gene [8]. The *Ph-3* QTL explained 41.5% of the phenotypic variation in the mapping population. The additive effect was −0.78, and the dominant effect was −0.26. This indicated that the L3708 allele increased resistance to the Pi39A isolate and led to a reduction in the DSR score when the L3708 allele replaced the allele from cultivated tomato. The L3708 allele is partially dominant to the allele from the cultivated tomato.

**Table 2:** List of QTLs for late blight resistance

inoculumisolate	location		marker name	LOD	PVE (%)	additiveeffect	dominanteffect
Pi39A	Chr.9	116.4cM	09g66864250	10.14	41.5	−0.78	−0.26
Pi733	Chr.2	13.7cM	02g30827526	8.18	11.9	−0.63	−0.31
Pi733	Chr.9	116.4 cM	09g66864250	25.44	63.3	−1.70	−0.18

Two QTLs were detected when the disease reaction of the aggressive isolate Pi733 was examined (Figure 3, Table 2 and S7). The QTL on chromosome 9 mapped to the same chromosomal region as that to the Pi39A isolate, so this QTL was also designated as the *Ph-3* gene. This *Ph-3* QTL explained 63.3% of the phenotypic variation. The additive effect was −1.70 and the dominant effect was −0.18. This indicated that the L3708 allele of *Ph-3* increased resistance to the isolate Pi733, and the effect of the L3708 allele of *Ph-3* was almost additive when L3708 allele replaced the allele from cultivated tomato. The second detected QTL was close to the proximal end of the centromere on chromosomal 2. This QTL was not identified in the first round of interval mapping analysis, but detected after the marker closely linked to the *Ph-3* gene was used as a cofactor in the MQM mapping analysis. This QTL has not been previously reported and was named *qPh2.1*. *qPh2.1* explained only 11.9% of the phenotypic variation. The additive effect was −0.63 and the dominant effect was −0.31. Therefore, the L3708 allele of *qPh2.1* was partially dominant to the cultivated tomato allele of *qPh2.1*. The L3708 allele of *qPh2.1* also confers resistance to the Pi733 isolate. When compared with the effect of *Ph-3* in the same mapping population, *qPh2.1* can be classified as a minor QTL.

## Discussion

### The RAD linkage genetic map

Two factors are major keys for SNP discovery using next generation sequencing technology. The first is to build correct alignment between reads and the reference sequences, the second is to generate a sufficient number of read sequences from the high-throughput sequencing platform [45]. The former can reduce the false positive rate, while the latter can reduce the false negative rate. For the RAD protocol, correct alignment is easier to achieve; this is because all RAD sequence reads have the same length (90bp after trimming barcode sequences and restriction site remains) and anchored at specific restriction sites (*PstI*), thus greatly reducing alignment ambiguity. Furthermore, correct SNP sites show normal genotypic segregation in the progenies. Therefore, it is easy to fix incorrect alignment problems using the RAD data. In contrast, an insufficient number of short read sequences resulting from the nature of RAD library constructions or lack of restriction sites in one parental line, is the major causes of missing data and genotyping errors [44].

To obtain a sufficient number of short read sequences for a sample in a cost-effective manner, four factors were considered before RAD libraries were generated: (1) the number of *PstI* restriction sites in the tomato genome; (2) the read depth for a given RAD site; (3) the number of barcoded samples pooled in a RAD library; and (4) the product of the aforementioned three factors was set equal to the total number of single-end short reads generated from one lane of the Illumina Hiseq2000 platform. We performed a conservative estimation for these factors as follows: (1) There are 82,814 *PstI* sites in tomato genome sequence build SL2.40, therefore 165,628 RAD sites were expected; (2) the average read depth for all RAD sites was set to nine, based on an estimation of 99% probability that the read depth of any RAD site is no less than three; (3) 60 barcoded tomato samples were pooled in a RAD library; (4) at least 89.4 million reads per lane were required to be generated from the Illumina Hiseq2000 platform, and each sample required at least 1.5 million reads in order to obtain correct marker genotypes. In this study, the Illumina service center was able to generate 198 million reads per lane. However, 12 samples had the read number less than 1.5 million. These samples also had at least 327 missing genotypes out of 4697 markers (Figure 2). To mitigate genotyping error problems [44], we set 327 missing genotypes as the threshold, and removed additional 12 samples whose number of missing genotypes greater than 327. Therefore, 96 samples were remained to construct the genetic linkage map.

The length of the genetic map in this study is longer than in previous studies [25], [46]. Inflation of genetic maps usually results from genotyping errors [47]. The same phenomena was observed for construction of an *Arabidopsis* genetic map in a backcross population using genotyping technology similar to RAD sequencing [30]. Because marker order can be affected by genotyping errors, we deployed a strategy to identify markers that resulted in the same marker order between the genetic and physical maps. Consequentially, the overall genotyping error rate for the 395 RAD markers was reduced to 2.18%, a percentage that would not seriously affect the QTL analysis results.

The genetic map in this study showed similar features to a typical tomato genetic map in which high recombination rate was found at the distal ends of all chromosomes, but recombination was suppressed in the pericentromeric regions [25], [48]. All VeraCode SNP markers in the pericentromeric regions showed identical genotypes for all individuals in the mapping population, therefore, markers on the region with identical genotype could be defined as the region that recombination was suppressed. Furthermore, the physical regions at which recombination was suppressed were similar to regions found in a previous study (Table S3) [25].

It is worth to note that our strategy to select RAD markers showing collinear orders between the physical map and the genetic map depends on how well the physical map was built. The higher accuracy the physical map was built, the more the collinear RAD markers could be obtained. The predicted tomato genomic size is 900 megabases (Mb), of which 760Mb were assembled into 91 scaffolds with most gaps restricted in the pericentromeric region where recombination were commonly suppressed [48]. While some RAD sites might locate at gaps outside the scaffolds, these RAD markers were removed early by filtration of potentially problematic markers. In addition, base accuracy of the tomato reference genome sequences is approximately one substitution error per 29.4 kb [48]. Given that 82,814 *PstI* sites were identified in the tomato reference genome, only 507 base substitution errors were expected as the false positive markers which were counted less than one tenth of overall genuine SNPs. Finally, a recent study which constructed a high density genetic map using the tomato high-throughput genotyping array with 7720 SNPs, demonstrated good collinearity between the genetic map and the physical map with few exceptional regions on chromosome 3, 10, and 12 [25]. The genetic map constructed in this study had medium marker density and was obviously not affected by those exceptional chromosomal regions. In conclusion, the tomato physical map is well built, so our strategy to select collinear RAD markers for genetic map construction is effective.

### QTLs associated with resistance to late blight

The major QTL associated with resistance to late blight in L3708 was identified in the segment between 66,536,514 and 67,494,653 bp on chromosome 9, with the most significant marker locating at 66,864,250 bp (Table 2). This QTL was assigned as the *Ph-3* gene because TG591, the closely linked marker for the *Ph-3* gene, is located at genomic position 66.794 Mb (http://solgenomics.net/) [8]. Furthermore, the *Ph-3* gene has recently been delimited within the chromosomal region from 66,714,091 to 66,825,552 bp [49].

In this study, it seems that the *Ph-3* gene is race non-specific, because the *Ph-3* QTL was identified in both inoculations of Pi39A and Pi733. Nevertheless, from the result that examined the physiological race for Pi39A and Pi733, both isolates cannot infected the differential hosts L3708 and CLN2037B both of which possess the homozygous *Ph-3* resistant allele (Table 1). Based on Flor's gene-for-gene theory [50], this result implied that both Pi39A and Pi733 isolates have the avirulent factor that is incompatible with the *Ph-3* resistant allele. Identification of the *Ph-3* QTL from inoculation of different isolates could result from the fact that the other parental line *S. lycopersicum* T3224 possesses the homozygous susceptible allele at the *Ph-3* locus and hence the resistant allele and the susceptible allele at the *Ph-3* locus were segregated in the F_2:3_ mapping population. Therefore, results in this study still support the conclusion in the previous study that the *Ph-3* gene is a race-specific gene [8].

The main purpose of this study was to reexamine the genetic components of resistance to late blight in wild tomato *S. pimpinellifolium* L3708, and to ascertain whether another gene contributes to its late blight resistance. The results revealed that the newly identified *qPh2.1* QTL confers resistance specifically against isolate Pi733. However, whether the *qPh2.1* QTL is able to hold off the highly aggressive pathogen that can defeat the *Ph-3* gene [15], or if the *qPh2.1* QTL merely enhances resistance against highly aggressive isolates, as shown in this and in Kim and Mutschler's work [12], remains to be fully determined.

## Supporting Information

Table S1 **Information of barcoded adapters used to construct RAD libraries.**
(XLSX)

Table S2 **Total read numbers for each individual in the mapping population.**
(XLSX)

Table S3 **VeraCode marker information and genotypes.**
(XLSX)

Table S4 **Disease reactions to** ***P. infestans*** **isolates Pi39A and Pi733.**
(XLSX)

Table S5 **RAD marker information and genotypes.**
(XLSX)

Table S6 **Genetic map information.**
(XLSX)

Table S7 **QTL mapping results.**
(XLSX)
